# Assessing and Supporting Mental Health Outcomes Among Adolescents in Urban Informal Settlements in Kenya and Uganda

**DOI:** 10.1192/j.eurpsy.2023.2101

**Published:** 2023-07-19

**Authors:** G. Mbithi, A. Abubakar

**Affiliations:** 1 Aga Khan University, Nairobi, Kenya

## Abstract

**Introduction:**

Mental health problems among adolescents have been on the rise particularly among adolescents in sub-Saharan Africa (SSA) due to impoverished living conditions and a high burden of chronic diseases including HIV/AIDs. COVID-19 pandemic has further exacerbated the risk and placed additional stress on adolescents’ mental health. While the burden might be high, there are fewer mental health services in the region.

**Objectives:**

To evaluate the psychological and mental well-being of adolescents living in and co co-design with civil society organizations (CSOs) interventions aimed at enhancing mental health and psychosocial well-being.

**Methods:**

Firstly, we conducted a formative phase to assess the burden of various mental health problems in Kenya. We conducted a cross-sectional survey in which we assessed the mental health status of 1541 adolescents using standardized tools. The participants comprised in and out of school adolescents, adolescents with disability, and those living with HIV/AIDS. Furthermore, we undertook a qualitative study through FGDs and KIIs to identify the factors contributing to mental health problems.

Secondly, we aim to adapt interventions that seek to promote mental health. Finally, we aim to implement effective mental health interventions targeting over 2000 adolescents living in Kenya’ informal settlements.

**Results:**

We found the prevalence of depression to be higher among out-of-school adolescents at 36.0% compared to school-going adolescents at 20.6%. Furthermore, out-of-school adolescents had statistically significantly higher anxiety scores as well at 27.7 % when compared to their school-going counterparts at 19.1%. In-school adolescents had a better quality of life scores, lower pandemic anxiety scores, and lower emotional scores compared to their out-of-school counterparts. Results from regression models indicated that being out of school, having a COVID-19 infection, having poor relationships with parents and peers, loneliness, and living in an unsafe neighborhood were factors associated with poor mental health outcomes. During the qualitative interviews, participants noted that COVID- 19 brought about financial stress, joblessness, led to early pregnancies, involvement in commercial sex work by adolescents, school dropouts, lead to stress, and depression among other issues.

**Image 3:**

**Figure:**
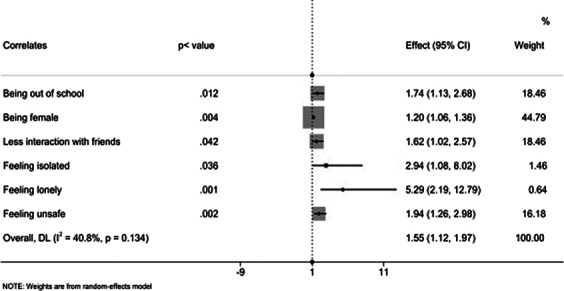

**Conclusions:**

Adolescents, especially those out of school are at a high risk of poor mental health outcomes due to exposure to a host of psychosocial risk factors. We have identified two interventions that we are keen to implement: the Shamiri Wellness Intervention (https://www.shamiri.institute/the-shamiri-intervention) and the Mental Health Literacy Programme (http://mentalhealthliteracy.org/). We hope that by working with CSOs, the study will support the development of their capacity to offer mental health services that are sustainable, and contextually appropriate.

**Disclosure of Interest:**

None Declared

